# Model-Free Adaptive Iterative Learning Bipartite Containment Control for Multi-Agent Systems

**DOI:** 10.3390/s22197115

**Published:** 2022-09-20

**Authors:** Shangyu Sang, Ruikun Zhang, Xue Lin

**Affiliations:** School of Mathematics and Physics, Qingdao University of Science and Technology, Qingdao 266061, China

**Keywords:** multi-agent systems, signed networks, model-free adaptive iterative learning control

## Abstract

This paper studies the bipartite containment tracking problem for a class of nonlinear multi-agent systems (MASs), where the interactions among agents can be both cooperative or antagonistic. Firstly, by the dynamic linearization method, we propose a novel model-free adaptive iterative learning control (MFAILC) to solve the bipartite containment problem of MASs. The designed controller only relies on the input and output data of the agent without requiring the model information of MASs. Secondly, we give the convergence condition that the containment error asymptotically converges to zero. The result shows that the output states of all followers will converge to the convex hull formed by the output states of leaders and the symmetric output states of leaders. Finally, the simulation verifies the effectiveness of the proposed method.

## 1. Introduction

Cooperative control problems of MASs have been widely studied [1,2,3]. As a fundamental topic of cooperative control of MASs, the containment control problem has been investigated in recent years. The containment control problem considers that there are multiple leaders in the network and all followers can converge to the convex hull formed by the leaders. Containment control algorithms for MASs with different dynamics have been proposed [4,5,6,7]. For example, for both continuous-time and discrete-time MASs, Liu et al. [4] presented the necessary and sufficient conditions which guarantee the achievement of containment control. Considering the heterogeneous agents, Zheng et al. [5] further studied the containment problem of heterogeneous MASs composed of first-order and second-order integrator agents. Moreover, the containment control problem for MASs with general linear dynamics has also been studied [6,7].

In the practical application, many industrial processes achieve the tasks in a repetitive environment, such as robot manipulators and injection molding process [8]. For systems that repeat the operation process over a finite time interval, the iterative learning control (ILC) was studied [9,10,11,12,13]. The control method uses the error between the current trajectory and the desired trajectory to improve the control performance. Then, it can achieve the desired trajectory tracking through learning from the repetitive tracking task. The ILC has been used for solving various cooperative problems of MASs [14,15,16,17,18,19]. Yang et al. [14] solved the consensus tracking problem of MASs with time-varying dynamics by the ILC method. Meng et al. [15] further studied the consensus tracking problem of MASs with initial state shifts and disturbances by the ILC method. In addition to the consensus tracking problem, the ILC has also been used to solve the formation control problem of MASs [17,18,19]. For example, Li et al. [18] proposed the distributed ILC protocols for solving the consensus and formation problem of second-order MASs.

Most of the above mentioned papers mainly used model-based control methods to design the controller. However, modeling a practical plant is an approximation of the real plant. As the complexity of system increases, it becomes more difficult to establish the accurate model. Even if an accurate model is established, it may be very complicated due to many parameters. This will make the controller be more complicated and less robust. Motivated by the above questions, for nonlinear systems, Hou [20] first proposed the model-free adaptive control (MFAC), which effectively avoids the disadvantages caused by the complexity of the real systems. The MFAC is a data-driven control method, which only uses input and output (I/O) data of systems to design the controller. The MFAC method has been employed to solve many control problems for discrete-time nonlinear systems due to its advantages [21,22,23]. Taking advantages of the MFAC and the ILC, the MFAILC method was proposed for the repeated operating systems for which it is difficult to establish the model. Recently, the MFAILC method has been applied for solving the control problems of MASs [24,25,26,27,28]. For example, Bu et al. [24] solved the consensus tracking problem of MASs under the fixed and the iteration-varying topologies by a MFAILC method. In contrast to [24], Wang et al. [25] used the MFAILC algorithm to solve the consensus tracking problem of MASs with unknown disturbance. For this problem, Zhao et al. [28] further analyzed the MASs with sensor saturation and measurement disturbance by the MFAILC algorithm. Considering the existence of multiple leaders, Hua et al. [27] studied the containment control problem of MASs with unknown nonlinear dynamics by the event-triggered MFAILC method. The aforementioned papers mainly consider the cooperative interaction among agents in the network. It is worthwhile to note that the antagonistic (e.g., the hostile or distrustful) interaction among individuals is ubiquitous [29,30]. Generally, the cooperative and antagonistic interactions among agents can be characterized by the signed network, where the negative (positive) edge represents the antagonistic (cooperative) interaction between agents. Altafini [29] pointed out that, if the signed network is structurally balanced, then the bipartite consensus can be achieved. Meng [31] further studied the bipartite containment tracking problem for linear systems. Additionally, there are many studies considering signed networks [32,33].

To the best of our knowledge, the MFAILC method is rarely considered for the bipartite containment tracking problem of MASs. In this paper, we will study the bipartite containment tracking problem for a class of nonlinear discrete-time MASs with unknown dynamics by the MFAILC method. In contrast to the linear systems considered in [31], in our paper, the nonlinear discrete-time MASs with unknown dynamics is considered, where each agent runs in a repetitive environment over a finite time interval. The main contributions are described as follows. First, for the model-free MASs, we establish a dynamical linear relationship between two iterations of each agent. That is, a dynamic linearization data model is equivalent to the initial nonlinear MASs. Second, by the dynamic linearization method, we propose a MFAILC. The controller design only needs I/O data of MASs and does not need other model information. Finally, we propose the condition for solving the bipartite containment problem of MASs. To verify the effectiveness of the MFAILC method, a simulation is given. The simulation shows that our proposed MFAILC method can solve the bipartite containment problem of MASs.

**Notation:** Let *R* and $R^{n\times n}$ represent the sets of real numbers and n×n-dimensional real matrices, respectively. $\mathrm{diag}\left(A\right)=\mathrm{diag}\left\{a_{11},a_{22},\ldots ,a_{nn}\right\}$ and *I* denotes the identity matrix with appropriate dimensions. ∥·∥ denotes 1-norm, and |·| represents the absolute value.

## 2. Preliminaries and Problem Statement

### 2.1. Preliminaries

**Figure 1:**
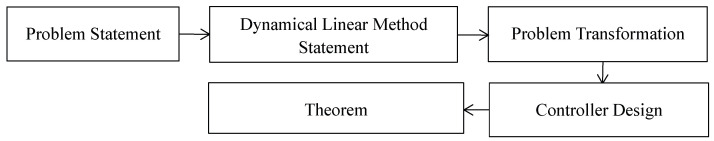
The flowchart of solving the bipartite containment problem of MASs.

Let G(V,E,A) be a signed network, where V={1,2,…,m+n}, E={(i,j)|i,j∈V}⊆V×V, $A=\left[a_{ij}\right]\in R^{\left(m+n)\times (m+n\right)}$ denotes the node set, the edge set, the adjacency matrix, respectively. $a_{ij}\neq 0$ if (j,i)∈E. (j,i) denotes the edge from *j* to *i*. $a_{ij}>0$ denotes the cooperative interaction between *i* and *j*, $a_{ij}<0$ denotes the antagonistic interaction, and $a_{ii}=0$ for all i∈V. The neighbor set of node *i* is depicted by $N_{i}=\left\{j|\left(j,i\right)\in E\right\}$. Let $V_{1}$ be a subset of V and $N_{V_{1}}=\left\{j\in V\backslash V_{1}|\exists i\in V_{1}:\left(j,i\right)\in E\right\}$, that is, $N_{V_{1}}$ represents the set of neighbors of the agent in $V_{1}$ such that these neighbors belong to the complement set of $V_{1}$. If node *i* is in a strongly connected subgraph $G_{s}$ of G(V,E,A) and $N_{G_{s}}=⌀$, node *i* can be called a leader, and *i* is called a follower otherwise. The Laplacian matrix $L=[l_{ij}]=D-A$, where $D=\mathrm{diag}\{d_{1},d_{2},\ldots ,d_{m+n}\}$ and $d_{i}=\sum_{j=1}^{m+n}\left|a_{ij}\right|$. G(V,E,A) is structurally balanced if it admits a bipartition of the nodes $V_{1}$, $V_{2}$, $V_{1}\cup V_{2}=V$, and $V_{1}\cap V_{2}=⌀$, such that $a_{ij}\geq 0$, $\forall i,j\in V_{k}\left(k\in \left\{1,2\right\}\right)$, and $a_{ij}\leq 0$, $\forall i\in V_{p},j\in V_{q},p\neq q\left(p,q\in \left\{1,2\right\}\right)$. It is said structurally unbalanced otherwise. Next, we will follow the flowchart in Figure 1 to solve the bipartite containment problem of MASs.

### 2.2. Problem Statement

We will discuss the bipartite containment control of the nonlinear discrete-time MASs by the MFAILC method. We consider *m* leaders and *n* followers in a signed network, where the *m* leaders are divided into groups $V_{1}$ and $V_{2}$, respectively. Assume that $V_{1}$ has $m_{1}$ leaders and $V_{2}$ has $m_{2}$ leaders, where $m=m_{1}+m_{2}$. The graph consisting of $m_{1}$ leaders is strongly connected and structurally balanced, that is, it admits a bipartition of the nodes $V_{1}^{\prime }$, $V_{1}^{\prime \prime }$, $V_{1}=V_{1}^{\prime }⋃V_{1}^{\prime \prime }$, and $V_{1}^{\prime }\cap V_{1}^{\prime \prime }=\emptyset$. The graph consisting of $m_{2}$ leaders is also strongly connected and structurally balanced, that is, it also admits a bipartition of the nodes $V_{2}^{\prime }$, $V_{2}^{\prime \prime }$, $V_{2}=V_{2}^{\prime }⋃V_{2}^{\prime \prime }$, and $V_{2}^{\prime }\cap V_{2}^{\prime \prime }=\emptyset$. The interactions among leaders include the antagonistic interaction. Moreover, the *n* followers are included in group $V_{3}$. The interactions among leaders and followers are cooperative, and the interactions among followers are also cooperative.

The dynamic of the *i*-th agent is written as

(1) $$\begin{array}{cc} y_{i}\left(N+1,\hbar \right)= & f_{i}(y_{i}\left(N,\hbar \right),y_{i}\left(N-1,\hbar \right),\ldots ,y_{i}\left(N-n_{y},\hbar \right),\\ \; & u_{i}\left(N,\hbar \right),u_{i}\left(N-1,\hbar \right),\ldots ,u_{i}\left(N-n_{u},\hbar \right)), \end{array}$$

where i∈{1,2,…,m+n} represents the *i*-th agent, N∈{0,1,…,T} represents time, and *ℏ* represents the iteration number. $u_{i}\left(N,\hbar \right)\in R$ and $y_{i}\left(N,\hbar \right)\in R$, respectively, represents the I/O of the *i*-th agent. $n_{y}$ and $n_{u}$, respectively, represents the unknown orders of *y* and *u*. $f_{i}\left(\cdot \right)$ represents the nonlinear function, and it only consists of the I/O of the *i*-th agent.

**Assumption** **1.** *G(V,E,A) is fixed and $\sum_{j=1}^{m+n}|a_{ij}|>\sum_{j=m+1}^{m+n}\left|a_{ji}\right|$ for any $i\in V_{3}$.*

**Assumption** **2.** *The partial derivative of $f_{i}\left(\cdot \right)$ with respect to $u_{i}\left(N,\hbar \right)$ is continuous, and $f_{i}\left(\cdot \right)$ satisfies the Lipschitz condition $\left|\Delta y_{i}\left(N+1,\hbar \right)\right|\leq b\left|\Delta u_{i}\left(N,\hbar \right)\right|$, where $\Delta y_{i}\left(N+1,\hbar \right)\overset{\Delta }{\;=}y_{i}\left(N+1,\hbar \right)-y_{i}\left(N+1,\hbar -1\right)$, $\Delta u_{i}\left(N,\hbar \right)\overset{\Delta }{\;=}u_{i}\left(N,\hbar \right)-u_{i}\left(N,\hbar -1\right)$, and b is a positive constant.*

**Lemma** **1 ([34]).** *If system (1) satisfies Assumption 2 and $\Delta u_{i}\left(N,\hbar \right)\neq 0$, then we have*

(2) $$\Delta y_{i}\left(N+1,\hbar \right)=\phi_{i}\left(N,\hbar \right)\Delta u_{i}\left(N,\hbar \right),$$

*where $\phi_{i}\left(N,\hbar \right)$ is a time-varying parameter associated with iteration, which is called pseudo partial derivative. $\phi_{i}\left(N,\hbar \right)\leq b^{\prime }$ and $b^{\prime }$ is a positive constant for any N and ℏ.*

Equation (2) can transform the nonlinear system of each agent into a linear dynamic system with a time-varying parameter and does not require the model information of MASs. The time-varying parameter $\phi_{i}\left(N,\hbar \right)$ can also be estimated only by the input and output data of MASs.

**Assumption** **3.** *For any time instant N and ℏ, the pseudo partial derivative satisfies $\phi_{i}\left(N,\hbar \right)>\sigma >0$ or $\phi_{i}\left(N,\hbar \right)<-\sigma <0$, where σ is an arbitrarily positive constant. Without loss of generality, we assume $\phi_{i}\left(N,\hbar \right)>\sigma >0$.*

Assumption 2 sets a bound on the change rate of the output caused by the change of input, which is a common situation in some real systems. Assumption 3 ensures that the increment of output and the increment of input have the same sign, which means that when the control input increases, the output must increase.

## 3. Main Result

In this part, we will show the controller and the condition in which the bipartite containment control is achieved.

We assume that agent *i*, i=1,2,…,m, is leader, and agent *i*, i=m+1,m+2,…,m+n, is follower. The subgraph composed of *m* leaders is $G_{L}$ and the subgraph composed of *n* followers is $G_{F}$. Then, the adjacency matrix of G(V,E,A) is written as

$$A=\left[\begin{array}{cc} A_{L} & 0_{m\times n}\\ A_{FL} & A_{F} \end{array}\right],$$

where $A_{L}$ denotes the adjacency matrix of $G_{L}$, $A_{F}$ denotes the adjacency matrix of $G_{F}$, and $A_{FL}$ denotes the interactions among leaders and followers. The Laplacian matrix of G(V,E,A) is written as

$$L=\left[\begin{array}{cc} L_{L} & 0_{m\times n}\\ -A_{FL} & L_{F}+D_{FL} \end{array}\right],$$

where $L_{L}$ and $L_{F}$ are Laplacian matrices associated with $A_{L}$ and $A_{F}$, respectively. $L_{F}=D_{FF}-A_{F}$, $D_{FF}=\mathrm{diag}\left\{d_{1},d_{2},\ldots ,d_{n}\right\}$ with $d_{i}=\sum_{j=m+1}^{m+n}\left|a_{(m+i)j}\right|$, 1≤i≤n, and $D_{FL}=\mathrm{diag}\left\{d_{1}^{\prime },d_{2}^{\prime },\ldots ,d_{n}^{\prime }\right\}$ with $d_{i}^{\prime }=\sum_{j=1}^{m}\left|a_{(m+i)j}\right|$, 1≤i≤n.

**Definition** **1.** *The containment error of the i-th agent at the ℏ-th iteration is denoted by $\xi_{i}\left(N,\hbar \right)$. For $i\in V_{1}⋃V_{2}$, we have*

(3) $$\xi_{i}\left(N,\hbar \right)=\sum_{j\in N_{i}}(a_{ij}y_{j}\left(N,\hbar \right)-|a_{ij}\left|y_{i}\left(N,\hbar \right)\right)+c_{i}\left(b_{i}y_{0}-y_{i}\left(N,\hbar \right)\right),$$

*where $y_{0}$ is a fixed constant, $y_{0}=y_{0}$ for $i\in V_{1}$, and $y_{0}=y_{0}^{\prime }$ for $i\in V_{2}$. $c_{i}\in \left\{0,1\right\}$, and $c_{i}=1$ denotes that agent i can receive the information $y_{0}$, otherwise $c_{i}=0$. We consider that there is at least one $c_{i}=1$ for $i\in V_{1}$, and there is at least one $c_{i}=1$ for $i\in V_{2}$. $b_{i}=1$ for $i\in V_{p}^{\prime }$, p∈{1,2}, and $b_{i}=-1$ for $i\in V_{p}^{\prime \prime }$, p∈{1,2}. For $i\in V_{3}$, we have*

(4) $$\xi_{i}\left(N,\hbar \right)=\sum_{j\in N_{i}}a_{ij}\left(y_{j}\left(N,\hbar \right)-y_{i}\left(N,\hbar \right)\right).$$

Containment errors of all agents in the form of vectors are written as

$$\xi \left(N,\hbar \right)={\left[\xi_{1}\left(N,\hbar \right),\xi_{2}\left(N,\hbar \right),\ldots ,\xi_{m+n}\left(N,\hbar \right)\right]}^{T}.$$

In order to facilitate the proof, we let $\xi \left(N,\hbar \right)={\left[\xi_{L}^{T}\left(N,\hbar \right),\xi_{F}^{T}\left(N,\hbar \right)\right]}^{T}$, where

(5) $$\xi_{L}\left(N,\hbar \right)={\left[\xi_{1}\left(N,\hbar \right),\xi_{2}\left(N,\hbar \right),\ldots ,\xi_{m}\left(N,\hbar \right)\right]}^{T},$$

(6) $$\xi_{F}\left(N,\hbar \right)={\left[\xi_{m+1}\left(N,\hbar \right),\xi_{m+2}\left(N,\hbar \right),\ldots ,\xi_{m+n}\left(N,\hbar \right)\right]}^{T}.$$

The vector Y(N,ℏ) is denoted by $Y\left(N,\hbar \right)={\left[y_{1}\left(N,\hbar \right),y_{2}\left(N,\hbar \right),\ldots ,y_{m+n}\left(N,\hbar \right)\right]}^{T}={\left[Y_{L}^{T}\left(N,\hbar \right),Y_{F}^{T}\left(N,\hbar \right)\right]}^{T}$, where

(7) $$Y_{L}\left(N,\hbar \right)={\left[y_{1}\left(N,\hbar \right),y_{2}\left(N,\hbar \right),\ldots ,y_{m}\left(N,\hbar \right)\right]}^{T},$$

(8) $$Y_{F}\left(N,\hbar \right)={\left[y_{m+1}\left(N,\hbar \right),y_{m+2}\left(N,\hbar \right),\ldots ,y_{m+n}\left(N,\hbar \right)\right]}^{T}.$$

By Equation (4), we can get

(9) $$\xi_{F}\left(N,\hbar \right)=A_{FL}Y_{L}\left(N,\hbar \right)-\left(L_{F}+D_{FL}\right)Y_{F}\left(N,\hbar \right).$$

Our goal is to design a controller for MASs, so that the bipartite containment can be achieved, that is, the output states $y_{i}\left(N,\hbar \right)$ of followers converge to the convex hull formed by the output states $y_{i}\left(N,\hbar \right)$ of leaders and the reverse output states $-y_{i}\left(N,\hbar \right)$ of leaders. If we can prove that $\lim_{\hbar \to \infty }y_{i}\left(N,\hbar \right)=y_{0}$ for $i\in V_{1}^{\prime }$, $\lim_{\hbar \to \infty }y_{i}\left(N,\hbar \right)=-y_{0}$ for $i\in V_{1}^{\prime \prime }$, $\lim_{\hbar \to \infty }y_{i}\left(N,\hbar \right)=y_{0}^{\prime }$ for $i\in V_{2}^{\prime }$, $\lim_{\hbar \to \infty }y_{i}\left(N,\hbar \right)=-y_{0}^{\prime }$ for $i\in V_{2}^{\prime \prime }$, and $\lim_{\hbar \to \infty }^{}∥\xi_{F}\left(N,\hbar \right)∥=0$, then the bipartite containment can be achieved. If $\lim_{\hbar \to \infty }^{}∥\xi_{F}\left(N,\hbar \right)∥=0$, then we have $\left(L_{F}+D_{FL}\right)\lim_{\hbar \to \infty }Y_{F}\left(N,\hbar \right)=A_{FL}\lim_{\hbar \to \infty }Y_{L}\left(N,\hbar \right)$. Since the graph consisting of *n* followers is strongly connected and $D_{FL}$ is a nonzero matrix, by the Gersgorin disc theorem and Theorem 6.2.26 in [35], we know that zero is not an eigenvalue of $L_{F}+D_{FL}$. Thus, we have $\lim_{\hbar \to \infty }Y_{F}\left(N,\hbar \right)={\left(L_{F}+D_{FL}\right)}^{-1}A_{FL}\lim_{\hbar \to \infty }Y_{L}\left(N,\hbar \right)$. Then, by Lemma 4 in [36], we know that ${\left(L_{F}+D_{FL}\right)}^{-1}A_{FL}$ is a nonnegative matrix and each row sum of ${\left(L_{F}+D_{FL}\right)}^{-1}A_{FL}$ is one. Then, the output states of followers converge to the convex hull formed by the output states and the reverse output states of leaders. Thus, we just need to prove that the leaders in $V_{1}$ and the leaders in $V_{2}$ achieve a bipartite consensus, and $\lim_{\hbar \to \infty }^{}∥\xi_{F}\left(N,\hbar \right)∥=0$.

The controller for every agent is designed as

(10) $$u_{i}\left(N,\hbar \right)=u_{i}\left(N,\hbar -1\right)+\frac{\rho {\hat{\phi }}_{i}\left(N,\hbar \right)}{\lambda +{\left|{\hat{\phi }}_{i}\left(N,\hbar \right)\right|}^{2}}\xi_{i}\left(N+1,\hbar -1\right),$$

where λ>0 represents the weighting factor that will effect the stability of MASs, and ρ∈(0,1) represents the parameter of controller (10) that will affect the convergence properties. ${\hat{\phi }}_{i}\left(N,\hbar \right)$ is the estimated value of $\phi_{i}\left(N,\hbar \right)$, and ${\hat{\phi }}_{i}\left(N,\hbar \right)$ is updated by

(11) $$\begin{array}{cc} {\hat{\phi }}_{i}\left(N,\hbar \right)= & {\hat{\phi }}_{i}\left(N,\hbar -1\right)+\frac{\eta \Delta u_{i}\left(N,\hbar -1\right)}{\mu +|\Delta u_{i}{\left(N,\hbar -1\right)|}^{2}}(\Delta y_{i}\left(N+1,\hbar -1\right)\\ \; & -{\hat{\phi }}_{i}\left(N,\hbar -1\right)\Delta u_{i}\left(N,\hbar -1\right)), \end{array}$$

where 0<η<1 and μ>0. The following equation is the reset condition which can ensure the robustness of controller:

(12) $${\hat{\phi }}_{i}\left(N,\hbar \right)={\hat{\phi }}_{i}\left(N,1\right),$$

which holds if any of the following three equations is satisfied $|{\hat{\phi }}_{i}\left(N,\hbar \right)|\leq \sigma ,$ $|\Delta u_{i}\left(N,\hbar -1\right)|\leq \sigma ,$ $\mathrm{or}\;\;\mathrm{sign}\left(\left({\hat{\phi }}_{i}\left(N,\hbar \right)\right)\right)\neq \mathrm{sign}\left(\left({\hat{\phi }}_{i}\left(N,1\right)\right)\right).$

**Remark** **1.** *Inspired by normalized least mean squares, we design the objective function $J\left({\hat{\phi }}_{i}\left(N,\hbar \right)\right)=|\Delta y_{i}\left(N+1,\hbar -1\right)-{\hat{\phi }}_{i}\left(N,\hbar \right)\Delta u_{i}{\left(N,\hbar -1\right)|}^{2}+\mu {\left|{\hat{\phi }}_{i}\left(N,\hbar \right)-{\hat{\phi }}_{i}\left(N,\hbar -1\right)\right|}^{2}$. By the optimization condition $\left(\partial J\left({\hat{\phi }}_{i}\left(N,\hbar \right)\right)\right)/\left(\partial {\hat{\phi }}_{i}\left(N,\hbar \right)\right)=0$, we can get (11). Similarly, we design the objective function $J\left(u_{i}\left(N,\hbar \right)\right)=|\xi_{i}\left(N+1,\hbar -1\right)-{\hat{\phi }}_{i}\left(N,\hbar \right)\Delta u_{i}{\left(N,\hbar \right)|}^{2}+\lambda {\left|\Delta u_{i}\left(N,\hbar \right)\right|}^{2}$. By the optimization condition $\left(\partial J\left(u_{i}\left(N,\hbar \right)\right)\right)/\left(\partial u_{i}\left(N,\hbar \right)\right)=0$, we can get (10).*

**Theorem** **1.** *Suppose that system (1) with controller (10) satisfies Assumptions 1−3. Then, $\lim_{\hbar \to \infty }y_{i}\left(N,\hbar \right)$ = $b_{i}y_{0}$ for $i\in V_{1}$, $\lim_{\hbar \to \infty }y_{i}\left(N,\hbar \right)=b_{i}y_{0}^{\prime }$ for $i\in V_{2}$, and $\lim_{\hbar \to \infty }∥\xi_{F}\left(N,\hbar \right)∥$ = 0 if*

$$\lambda >\frac{{\left(b^{\prime }\right)}^{2}}{4}\;\;\mathrm{and}\;\;0<\rho \leq \frac{1}{\max_{i=1,2,\ldots ,m+n}(\sum_{j=1}^{m+n}\left|a_{ij}\right|+c_{i})}.$$

**Proof.**  First, we prove the boundedness of ${\hat{\phi }}_{i}\left(N,\hbar \right)$. Obviously, if ${\hat{\phi }}_{i}\left(N,\hbar \right)$ satisfies Equation (12), the boundedness of ${\hat{\phi }}_{i}\left(N,\hbar \right)$ can be ensured. Then, we prove that, when Equation (12) does not hold, the boundedness of ${\hat{\phi }}_{i}\left(N,\hbar \right)$ still holds. Let ${\tilde{\phi }}_{i}\left(N,\hbar \right)={\hat{\phi }}_{i}\left(N,\hbar \right)-\phi_{i}\left(N,\hbar \right)$ represent the parameter estimation error. By Equations (2) and (11), we have

(13) $$\begin{array}{cc} \tilde{\phi_{i}}\left(N,\hbar \right)= & \tilde{\phi_{i}}\left(N,\hbar -1\right)+\hat{\phi_{i}}\left(N,\hbar \right)\\ \; & -\phi_{i}\left(N,\hbar \right)-\hat{\phi_{i}}\left(N,\hbar -1\right)+\phi_{i}\left(N,\hbar -1\right)\\ = & \tilde{\phi_{i}}\left(N,\hbar -1\right)+\frac{\eta \Delta u_{i}\left(N,\hbar -1\right)}{\mu +|\Delta u_{i}{\left(N,\hbar -1\right)|}^{2}}\\ \; & \times (\phi_{i}\left(N,\hbar -1\right)\Delta u_{i}\left(N,\hbar -1\right)\\ \; & -{\hat{\phi }}_{i}\left(N,\hbar -1\right)\Delta u_{i}\left(N,\hbar -1\right))+(\phi_{i}\left(N,\hbar -1\right)\\ \; & -\phi_{i}\left(N,\hbar \right))\\ = & \left(1-\frac{\eta |\Delta u_{i}{\left(N,\hbar -1\right)|}^{2}}{\mu +|\Delta u_{i}{\left(N,\hbar -1\right)|}^{2}}\right)\tilde{\phi_{i}}\left(N,\hbar -1\right)\\ \; & +(\phi_{i}\left(N,\hbar -1\right)-\phi_{i}\left(N,\hbar \right)). \end{array}$$

By Lemma 1, we have $\phi_{i}\left(N,\hbar \right)\leq b^{\prime }$. Then

(14) $$\phi_{i}\left(N,\hbar -1\right)-\phi_{i}\left(N,\hbar \right)\leq 2b^{\prime }.$$

By 0<η<1 and μ>0, we have that there is a constant *q* satisfying

(15) $$0<1-\frac{\eta |\Delta u_{i}{\left(N,\hbar -1\right)|}^{2}}{\mu +{\left|\Delta u_{i}\left(N,\hbar -1\right)\right|}^{2}}\leq q<1.$$

By Equations (14) and (15), Equation (13) can be written as ${\tilde{\phi }}_{i}\left(N,\hbar \right)\leq q{\tilde{\phi }}_{i}\left(N,\hbar -1\right)+2b^{\prime }\leq \ldots \leq q^{\hbar -1}{\tilde{\phi }}_{i}\left(N,1\right)+\frac{2b^{\prime }\left(1-q^{\hbar -1}\right)}{1-q}.$ Since 0<q<1, we have $\lim_{\hbar \to \infty }q^{\hbar -1}=0$. Thus, ${\tilde{\phi }}_{i}\left(N,\hbar \right)$ is bounded. Since $\phi_{i}\left(N,\hbar \right)$ is bounded and ${\tilde{\phi }}_{i}\left(N,\hbar \right)={\hat{\phi }}_{i}\left(N,\hbar \right)-\phi_{i}\left(N,\hbar \right)$, we conclude that ${\hat{\phi }}_{i}\left(N,\hbar \right)$ is bounded.

In order to facilitate the following proof, we consider $\Delta u_{i}\left(N,\hbar \right)$. By Equation (10), we have

(16) $$\Delta u_{i}\left(N,\hbar \right)=\frac{\rho {\hat{\phi }}_{i}\left(N,\hbar \right)}{\lambda +{\left|{\hat{\phi }}_{i}\left(N,\hbar \right)\right|}^{2}}\xi_{i}\left(N+1,\hbar -1\right).$$

Then, we have

(17) ϕ(N,ℏ)Δu(N,ℏ)=ρHξ(N+1,ℏ−1),

where $H=\mathrm{diag}\{h_{1},h_{2},\ldots ,h_{m+n}\}$ with $h_{i}$ =$\frac{\phi_{i}\left(N,\hbar \right){\hat{\phi }}_{i}\left(N,\hbar \right)}{\lambda +{\left|{\hat{\phi }}_{i}\left(N,\hbar \right)\right|}^{2}}$, $\phi \left(N,\hbar \right)=\mathrm{diag}\{\phi_{1}\left(N,\hbar \right),\phi_{2}\left(N,\hbar \right),$$\ldots ,\phi_{m+n}\left(N,\hbar \right)\}$, and $\Delta u\left(N,\hbar \right)={\left[\Delta u_{1}\left(N,\hbar \right),\Delta u_{2}\left(N,\hbar \right),\ldots ,\Delta u_{m+n}\left(N,\hbar \right)\right]}^{T}$. By the Cauchy-Schwarz inequality, we have $\lambda +{\left|{\hat{\phi }}_{i}\left(N,\hbar \right)\right|}^{2}\geq 2\sqrt{\lambda }\left|{\hat{\phi }}_{i}\left(N,\hbar \right)\right|$. Since $\lambda >\frac{{\left(b^{\prime }\right)}^{2}}{4}$, then we have

(18) $$0<h_{i}\leq \frac{\phi_{i}\left(N,\hbar \right){\hat{\phi }}_{i}\left(N,\hbar \right)}{2\sqrt{\lambda }\left|{\hat{\phi }}_{i}\left(N,\hbar \right)\right|}\leq \frac{b^{\prime }}{2\sqrt{\lambda }}<1.$$

From Assumption 3, we know that $\phi_{i}\left(N,\hbar \right)$ is non-negative. By Equation (12), we can obtain that the sign of ${\hat{\phi }}_{i}\left(N,\hbar \right)$ is the same as the sign of ${\hat{\phi }}_{i}\left(N,1\right)$. As the estimated value of $\phi_{i}\left(N,\hbar \right)$, the nonnegativity of ${\hat{\phi }}_{i}\left(N,\hbar \right)$ can be guaranteed by choosing the initial value ${\hat{\phi }}_{i}\left(N,1\right)$ of ${\hat{\phi }}_{i}\left(N,\hbar \right)$.

Next, we will prove that leaders in group $V_{1}$ can achieve the bipartite consensus. Since $G(V_{1},E_{1},A_{1})$ is structurally balanced, $V_{1}$ can be divided into $V_{1}^{\prime }$ and $V_{1}^{\prime \prime }$ such that $a_{ij}\geq 0$, for any $i,j\in V_{1}^{\prime }$ or $i,j\in V_{1}^{\prime \prime }$, $a_{ij}\leq 0$, for any $i\in V_{1}^{\prime },j\in V_{1}^{\prime \prime }$. For *i*-th leader in $V_{1}$, by Equation (3), we have

(19) $$\begin{array}{cc} \xi_{i}\left(N,\hbar \right)= & \sum_{j\in N_{i}}[a_{ij}y_{j}\left(N,\hbar \right)-\left|a_{ij}\right|y_{i}\left(N,\hbar \right)+|a_{ij}|b_{i}y_{0}-|a_{ij}\left|b_{i}y_{0}\right]+c_{i}e_{i}\left(N,\hbar \right)\\ = & \sum_{j\in N_{i}}\left[\right|a_{ij}|e_{i}\left(N,\hbar \right)+a_{ij}y_{j}\left(N,\hbar \right)-|a_{ij}\left|b_{i}y_{0}\right]+c_{i}e_{i}\left(N,\hbar \right), \end{array}$$

where $e_{i}\left(N,\hbar \right)=b_{i}y_{0}-y_{i}\left(N,\hbar \right)$, $b_{i}=1$ for any $i\in V_{1}^{\prime }$, and $b_{i}=-1$ for any $i\in V_{1}^{\prime \prime }$. Then, Equation (19) is written as

(20) $$\begin{array}{cc} \xi_{i}\left(N,\hbar \right)= & \sum_{j\in N_{i}}\left[\right|a_{ij}\left|e_{i}\left(N,\hbar \right)+a_{ij}y_{j}\left(N,\hbar \right)-a_{ij}b_{j}y_{0}\right]+c_{i}e_{i}\left(N,\hbar \right)\\ = & \sum_{j\in N_{i}}\left[\right|a_{ij}\left|e_{i}\left(N,\hbar \right)-a_{ij}e_{j}\left(N,\hbar \right)\right]+c_{i}e_{i}\left(N,\hbar \right). \end{array}$$

Then, we have

(21) $${\bar{\xi }}_{1}\left(N,\hbar \right)=\left(L_{1}+C\right)e\left(N,\hbar \right),$$

where ${\bar{\xi }}_{1}\left(N,\hbar \right)={\left[\xi_{1}\left(N,\hbar \right),\xi_{2}\left(N,\hbar \right),\ldots ,\xi_{m_{1}}\left(N,\hbar \right)\right]}^{T}$, $C=\mathrm{diag}\{c_{1},c_{2},\ldots ,c_{m_{1}}\}$, $L_{1}$ is the Laplacian matrix of $G(V_{1},E_{1},A_{1})$, and $e\left(N,\hbar \right)={\left[e_{1}\left(N,\hbar \right),e_{2}\left(N,\hbar \right),\ldots ,e_{m_{1}}\left(N,\hbar \right)\right]}^{T}$.

Let Δe(N,ℏ)=e(N,ℏ)−e(N,ℏ−1). By $e_{i}\left(N,\hbar \right)=b_{i}y_{0}-y_{i}\left(N,\hbar \right)$, we have

(22) $$\Delta \bar{y}\left(N+1,\hbar \right)=-\Delta e\left(N+1,\hbar \right),$$

where $\Delta \bar{y}\left(N+1,\hbar \right)={\left[\Delta y_{1}\left(N+1,\hbar \right),\Delta y_{2}\left(N+1,\hbar \right),\ldots ,\Delta y_{m_{1}}\left(N+1,\hbar \right)\right]}^{T}$. By Equations (2), (17) and (21), we have

(23) $$\begin{array}{cc} e(N+1,\hbar )= & \Delta e(N+1,\hbar )+e(N+1,\hbar -1)\\ = & -\Delta \bar{y}\left(N+1,\hbar \right)+e\left(N+1,\hbar -1\right)\\ = & -\phi_{V_{1}}\left(N,\hbar \right)\Delta \bar{u}\left(N,\hbar \right)+e\left(N+1,\hbar -1\right)\\ = & \left(I-\rho H_{1}\left(N,\hbar \right)\left(L_{1}+C\right)\right)e\left(N+1,\hbar -1\right), \end{array}$$

where $H_{1}\left(N,\hbar \right)=\mathrm{diag}\left\{h_{1}\left(N,\hbar \right),h_{2}\left(N,\hbar \right),\ldots ,h_{m_{1}}\left(N,\hbar \right)\right\}$ with $h_{i}\left(N,\hbar \right)=\frac{\phi_{i}\left(N,\hbar \right){\hat{\phi }}_{i}\left(N,\hbar \right)}{\lambda +{\left|{\hat{\phi }}_{i}\left(N,\hbar \right)\right|}^{2}}$, $1\leq i\leq m_{1}$, $\Delta \bar{u}\left(N,\hbar \right)={\left[\Delta u_{1}\left(N,\hbar \right),\Delta u_{2}\left(N,\hbar \right),\ldots ,\Delta u_{m_{1}}\left(N,\hbar \right)\right]}^{T}$ and $\phi_{V_{1}}\left(N,\hbar \right)=\mathrm{diag}\left\{\phi_{1}\left(N,\hbar \right),\phi_{2}\left(N,\hbar \right),\ldots ,\phi_{m_{1}}\left(N,\hbar \right)\right\}$. Since $G(V_{1},E_{1},A_{1})$ is structurally balanced, by Lemma 1 in [29], there is *M* such that $MA_{1}M\geq 0$, where $M=\mathrm{diag}\{\tau_{1},\tau_{2},\ldots ,\tau_{m_{1}}\}$ and $\tau_{i}\in \left\{1,-1\right\}$. Let e(N+1,ℏ)=MZ(N+1,ℏ), by Equation (23), we have

(24) $$MZ\left(N+1,\hbar \right)=\left(I-\rho H_{1}\left(N,\hbar \right)\left(L_{1}+C\right)\right)MZ\left(N+1,\hbar -1\right).$$

Since $M^{-1}=M$, we have

(25) $$\begin{array}{c} Z\left(N+1,\hbar \right)=(I-\rho H_{1}\left(N,\hbar \right)L_{M})Z\left(N+1,\hbar -1\right), \end{array}$$

where $L_{M}=M\left(L_{1}+C\right)M$. Since $\rho \leq \frac{1}{\max_{i=1,2,\ldots m+n}(\sum_{j=1}^{m+n}\left|a_{ij}\right|+c_{i})}$, and $G(V_{1},E_{1},A_{1})$ is strongly connected, then matrix $I-\rho H_{1}\left(N,\hbar \right)L_{M}$ is nonnegative and irreducible. Since there is at least one $c_{i}=1$, then at least one row sum of $I-\rho H_{1}\left(N,\hbar \right)L_{M}$ is strictly less than one. Thus, $I-\rho H_{1}\left(N,\hbar \right)L_{M}$ is an irreducible substochastic matrix. By Equation (25), we have

(26) $$\begin{array}{cc} \; & ∥Z(N+1,\hbar )∥\\ \; & \leq ∥(I-\rho H_{1}\left(N,\hbar \right)L_{M})∥∥Z\left(N+1,\hbar -1\right)∥\\ \; & \vdots \\ \; & \leq ∥\left(I-\rho H_{1}\left(N,\hbar \right)L_{M}\right)∥∥\left(I-\rho H_{1}\left(N,\hbar -1\right)L_{M}\right)∥\\ \; & \ldots ∥(I-\rho H_{1}\left(N,2\right)L_{M})∥∥Z\left(N+1,1\right)∥. \end{array}$$

ω matrices chosen from $\left\{I-\rho H_{1}\left(N,\hbar \right)L_{M},I-\rho H_{1}\left(N,\hbar -1\right)L_{M},\ldots ,I-\rho H_{1}\left(N,2\right)L_{M}\right\}$ are multiplied together in (26), then by Lemma 1 in [37], we have

$$∥Z\left(N+1,\hbar \right)∥\leq \delta^{\left\lfloor \frac{\hbar -1}{\omega }\right\rfloor }∥Z\left(N+1,1\right)∥,$$

where $\left\lfloor \frac{\hbar -1}{\omega }\right\rfloor$ denotes the integer which is smaller than $\frac{\hbar -1}{\omega }$ and closest to $\frac{\hbar -1}{\omega }$, and 0<δ<1. By 0<δ<1, we have $\lim_{\hbar \to \infty }∥Z\left(N+1,\hbar \right)∥=0.$ By e(N+1,ℏ)=MZ(N+1,ℏ), we have $\lim_{\hbar \to \infty }∥e\left(N+1,\hbar \right)∥=0,$ which means that leaders in group $V_{1}$ achieve bipartite consensus. That is, $\lim_{\hbar \to \infty }y_{i}\left(N,\hbar \right)=y_{0}$ for $i\in V_{1}^{\prime }$ and $\lim_{\hbar \to \infty }y_{i}\left(N,\hbar \right)=-y_{0}$ for $i\in V_{1}^{\prime \prime }$. Similarly, leaders in group $V_{2}$ can also achieve bipartite consensus.

Next, we prove that $\lim_{\hbar \to \infty }∥\xi_{F}\left(N,\hbar \right)∥=0$. By Equations (2) and (8), we have

(27) $$Y_{F}\left(N+1,\hbar \right)=Y_{F}\left(N+1,\hbar -1\right)+\phi_{F}\left(N,\hbar \right)\Delta u_{F}\left(N,\hbar \right),$$

where $\phi_{F}\left(N,\hbar \right)=\mathrm{diag}\left\{\phi_{m+1}\left(N,\hbar \right),\phi_{m+2}\left(N,\hbar \right),\ldots ,\phi_{m+n}\left(N,\hbar \right)\right\}$ and $\Delta u_{F}\left(N,\hbar \right)$ =

$[\Delta u_{m+1}\left(N,\hbar \right),$$\Delta u_{m+2}\left(N,\hbar \right),\ldots ,\Delta u_{m+n}{\left(N,\hbar \right)]}^{T}$.

Let $D_{F}=D_{FF}+D_{FL}$. By Equation (9), we have

(28) $$\xi_{F}\left(N,\hbar \right)=A_{FL}Y_{L}\left(N,\hbar \right)+A_{F}Y_{F}\left(N,\hbar \right)-D_{F}Y_{F}\left(N,\hbar \right).$$

Multiply both sides of Equation (27) by $-D_{F}$, and add $A_{FL}Y_{L}\left(N+1,\hbar \right)+A_{F}Y_{F}\left(N+1,\hbar \right)+A_{FL}Y_{L}\left(N+1,\hbar -1\right)+A_{F}Y_{F}\left(N+1,\hbar -1\right)$ to both sides of Equation (27), we have

(29) $$\begin{array}{cc} \; & A_{FL}Y_{L}\left(N+1,\hbar \right)\;+\;A_{F}Y_{F}\left(N+1,\hbar \right)\;+\;A_{FL}Y_{L}\left(N+1,\hbar -1\right)\\ \; & +A_{F}Y_{F}\left(N+1,\hbar -1\right)-D_{F}Y_{F}\left(N+1,\hbar \right)\\ \; & =A_{FL}Y_{L}\left(N+1,\hbar \right)+A_{F}Y_{F}\left(N+1,\hbar \right)\\ \; & +A_{FL}Y_{L}\left(N+1,\hbar -1\right)+A_{F}Y_{F}\left(N+1,\hbar -1\right)\\ \; & -D_{F}Y_{F}\left(N+1,\hbar -1\right)-D_{F}\phi_{F}\left(N,\hbar \right)\Delta u_{F}\left(N,\hbar \right). \end{array}$$

By Equations (28) and (29), we have

(30) $$\begin{array}{cc} \; & \xi_{F}\left(N+1,\hbar \right)\;+\;A_{FL}Y_{L}\left(N+1,\hbar -1\right)\;+\;A_{F}Y_{F}\left(N+1,\hbar -1\right)\\ = & \xi_{F}\left(N+1,\hbar -1\right)+A_{FL}Y_{L}\left(N+1,\hbar \right)+A_{F}Y_{F}\left(N+1,\hbar \right)\\ \; & -D_{F}\phi_{F}\left(N,\hbar \right)\Delta u_{F}\left(N,\hbar \right). \end{array}$$

It follows from Equation (30) that

(31) $$\begin{array}{cc} \; & \xi_{F}\left(N+1,\hbar \right)\\ = & \xi_{F}\left(N+1,\hbar -1\right)+A_{FL}\Delta Y_{L}\left(N+1,\hbar \right)\\ \; & +A_{F}\Delta Y_{F}\left(N+1,\hbar \right)-D_{F}\phi_{F}\left(N,\hbar \right)\Delta u_{F}\left(N,\hbar \right), \end{array}$$

where $\Delta Y_{L}\left(N+1,\hbar \right)={\left[\Delta y_{1}\left(N+1,\hbar \right),\Delta y_{2}\left(N+1,\hbar \right),\ldots ,\Delta y_{m}\left(N+1,\hbar \right)\right]}^{T}$ and $\Delta Y_{F}\left(N+1,\hbar \right)={\left[\Delta y_{m+1}\left(N+1,\hbar \right),\Delta y_{m+2}\left(N+1,\hbar \right),\ldots ,\Delta y_{m+n}\left(N+1,\hbar \right)\right]}^{T}$. By Equation (27), we have

(32) $$\Delta Y_{F}\left(N+1,\hbar \right)=\phi_{F}\left(N,\hbar \right)\Delta u_{F}\left(N,\hbar \right).$$

Substituting (32) into (31), and by Equation (17), we have

(33) $$\begin{array}{cc} \; & \xi_{F}\left(N+1,\hbar \right)\\ = & \xi_{F}\left(N+1,\hbar -1\right)+A_{FL}\Delta Y_{L}\left(N+1,\hbar \right)\\ \; & +\left(A_{F}-D_{F}\right)\phi_{F}\left(N,\hbar \right)\Delta u_{F}\left(N,\hbar \right)\\ = & \xi_{F}\left(N+1,\hbar -1\right)-\left(L_{F}+D_{FL}\right)\phi_{F}\left(N,\hbar \right)\Delta u_{F}\left(N,\hbar \right)\\ \; & +A_{FL}\Delta Y_{L}\left(N+1,\hbar \right)\\ = & \left[I-\rho \left(L_{F}+D_{FL}\right)H_{F}\right]\xi_{F}\left(N+1,\hbar -1\right)\\ \; & +A_{FL}\Delta Y_{L}\left(N+1,\hbar \right), \end{array}$$

where $H_{F}=\mathrm{diag}\left\{h_{m+1},h_{m+2},\ldots ,h_{m+n}\right\}$ with $h_{i}=\frac{\phi_{i}\left(N,\hbar \right){\hat{\phi }}_{i}\left(N,\hbar \right)}{\lambda +{\left|{\hat{\phi }}_{i}\left(N,\hbar \right)\right|}^{2}}$, m+1≤i≤m+n.

By Equation (18), we have $0<h_{i}<1$. The 1-norm of matrix $I-\rho \left(L_{F}+D_{FL}\right)H_{F}$ can be written as $\max_{i}\left\{1-\rho h_{i}\left(\sum_{j=1}^{m+n}\left|a_{ij}\right|-\sum_{j=m+1}^{m+n}\left|a_{ji}\right|\right)\right\}.$ By Assumption 1, we know $\sum_{j=1}^{m+n}\left|a_{ij}\right|>\sum_{j=m+1}^{m+n}\left|a_{ji}\right|>0,$ then by $\rho \leq \frac{1}{\max_{i=1,\ldots ,m+n}\left(\sum_{j=1}^{m+n}\left|a_{ij}\right|+c_{i}\right)}$, we have $0<\sum_{j=1}^{m+n}\left|a_{ij}\right|-\sum_{j=m+1}^{m+n}\left|a_{ji}\right|<\frac{1}{\rho }$.

Then, $0<1-\rho h_{i}\left(\sum_{j=1}^{m+n}\left|a_{ij}\right|-\sum_{j=m+1}^{m+n}\left|a_{ji}\right|\right)<1.$ Thus, we have

$$0<∥I-\rho \left(L_{F}+D_{FL}\right)H_{F}∥\leq p<1,$$

where 0<p<1. Since leaders in both $V_{1}$ and $V_{2}$ achieve bipartite consensus, we have $\lim_{\hbar \to \infty }\Delta Y_{L}\left(N+1,\hbar \right)=0$. By Equation (33), we obtain

(34) $$\begin{array}{cc} \; & \xi_{F}\left(N+1,\hbar \right)\\ = & {\left[I-\rho \left(L_{F}+D_{FL}\right)H_{F}\right]}^{\hbar -\hbar_{0}}\xi_{F}\left(N+1,\hbar_{0}\right)\\ \; & +\sum_{i=\hbar_{0}+1}^{\hbar }{\left[I-\rho \left(L_{F}+D_{FL}\right)H_{F}\right]}^{\hbar -i}A_{FL}\Delta Y_{L}\left(N+1,i\right), \end{array}$$

where $1\leq \hbar_{0}\leq \hbar$. By $∥I-\rho \left(L_{F}+D_{FL}\right)H_{F}∥\leq p<1$, we have

$$\begin{array}{cc} ∥\xi_{F}\left(N+1,\hbar \right)∥ & \leq p^{\hbar -\hbar_{0}}∥\xi_{F}\left(N+1,\hbar_{0}\right)∥+∥A_{FL}∥\underset{\hbar_{0}\leq \tau \leq \hbar }{\sup}∥\Delta Y_{L}\left(N+1,\tau \right)∥\sum_{i=\hbar_{0}+1}^{\hbar }p^{\hbar -i}\\ & \leq p^{\hbar -\hbar_{0}}∥\xi_{F}\left(N+1,\hbar_{0}\right)∥+\frac{∥A_{FL}∥}{1-p}\underset{\hbar_{0}\leq \tau \leq \hbar }{\sup}∥\Delta Y_{L}\left(N+1,\tau \right)∥. \end{array}$$

Then, by $\lim_{\hbar \to \infty }\Delta Y_{L}\left(N+1,\hbar \right)$ = 0 and Lemma 4.7 in [38], we have $\lim_{\hbar \to \infty }∥\xi_{F}\left(N,\hbar \right)∥$=0. □

## 4. Simulation

**Figure 2:**
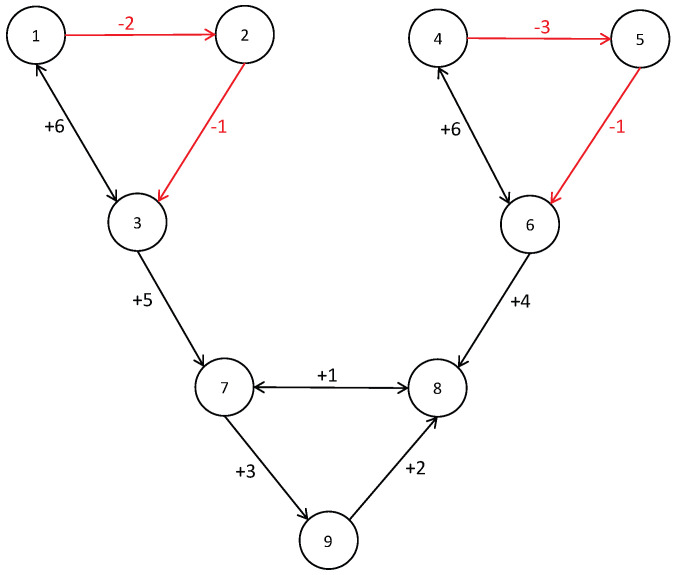
Signed graph.

We consider a MAS which consists of 6 leaders and 3 followers. The signed graph is shown in Figure 2 1–6 are leaders, 7–9 are followers. Moreover, the subgraph consisting of leaders 1,2,3 and the subgraph consisting of leaders 4,5,6 are structurally balanced. By the definition of adjacency matrix, we have the adjacency matrix of the signed graph shown in Figure 2 as follows

$$A=\left[\begin{array}{ccccccccc} 0 & 0 & 6 & 0 & 0 & 0 & 0 & 0 & 0\\ -2 & 0 & 0 & 0 & 0 & 0 & 0 & 0 & 0\\ 6 & -1 & 0 & 0 & 0 & 0 & 0 & 0 & 0\\ 0 & 0 & 0 & 0 & 0 & 6 & 0 & 0 & 0\\ 0 & 0 & 0 & -3 & 0 & 0 & 0 & 0 & 0\\ 0 & 0 & 0 & 6 & -1 & 0 & 0 & 0 & 0\\ 0 & 0 & 5 & 0 & 0 & 0 & 0 & 1 & 0\\ 0 & 0 & 0 & 0 & 0 & 4 & 1 & 0 & 2\\ 0 & 0 & 0 & 0 & 0 & 0 & 3 & 0 & 0 \end{array}\right].$$

By the definition of Laplacian matrix, we have the Laplacian matrix of signed graph shown in Figure 2 as follows

$$L=\left[\begin{array}{ccccccccc} 6 & 0 & -6 & 0 & 0 & 0 & 0 & 0 & 0\\ 2 & 2 & 0 & 0 & 0 & 0 & 0 & 0 & 0\\ -6 & 1 & 7 & 0 & 0 & 0 & 0 & 0 & 0\\ 0 & 0 & 0 & 6 & 0 & -6 & 0 & 0 & 0\\ 0 & 0 & 0 & 3 & 3 & 0 & 0 & 0 & 0\\ 0 & 0 & 0 & -6 & 1 & 7 & 0 & 0 & 0\\ 0 & 0 & -5 & 0 & 0 & 0 & 6 & -1 & 0\\ 0 & 0 & 0 & 0 & 0 & -4 & -1 & 7 & -2\\ 0 & 0 & 0 & 0 & 0 & 0 & -3 & 0 & 3 \end{array}\right].$$

Let ${\hat{\phi }}_{i}\left(N,1\right)=0.2,$ $u_{i}\left(N,1\right)=0$ and $Y\left(N,1\right)={\left[1,-0.2,2,-0.4,0.7,2,0,0,0\right]}^{T}$, where i∈{1,2,…,9}, N∈{1,2,…,20}, and $y_{0}=\frac{1}{2}$, $y_{0}^{\prime }=1$, $c_{1}=1$, $c_{4}=1$, and $c_{i}=0$ for i=2,3,5,6. The dynamic of the *i*-th (i=1,2,…,9) agent is written as

$$y_{1}\left(N+1,\hbar +1\right)=\frac{y_{1}\left(N,\hbar +1\right)+u_{1}\left(N,\hbar +1\right)}{1+y_{1}\left(N,\hbar +1\right)}+u_{1}\left(N,\hbar +1\right),$$

$$y_{2}\left(N+1,\hbar +1\right)=\frac{y_{2}\left(N,\hbar +1\right)+u_{2}\left(N,\hbar +1\right)}{1+y_{2}\left(N,\hbar +1\right)}+2\cdot u_{2}\left(N,\hbar +1\right),$$

$$y_{3}\left(N+1,\hbar +1\right)=\frac{y_{3}\left(N,\hbar +1\right)+u_{3}\left(N,\hbar +1\right)}{1+y_{3}\left(N,\hbar +1\right)}+3\cdot u_{3}\left(N,\hbar +1\right),$$

$$y_{4}\left(N+1,\hbar +1\right)=\frac{y_{4}\left(N,\hbar +1\right)+u_{4}\left(N,\hbar +1\right)}{1+y_{4}\left(N,\hbar +1\right)}+4\cdot u_{4}\left(N,\hbar +1\right),$$

$$y_{5}\left(N+1,\hbar +1\right)=\frac{y_{5}\left(N,\hbar +1\right)+u_{5}\left(N,\hbar +1\right)}{1+y_{5}\left(N,\hbar +1\right)}+5\cdot u_{5}\left(N,\hbar +1\right),$$

$$y_{6}\left(N+1,\hbar +1\right)=\frac{y_{6}\left(N,\hbar +1\right)+u_{6}\left(N,\hbar +1\right)}{1+y_{6}\left(N,\hbar +1\right)}+6\cdot u_{6}\left(N,\hbar +1\right),$$

$$y_{7}\left(N+1,\hbar +1\right)=\frac{y_{7}\left(N,\hbar +1\right)+u_{7}\left(N,\hbar +1\right)}{1+y_{7}\left(N,\hbar +1\right)}+7\cdot u_{7}\left(N,\hbar +1\right),$$

$$y_{8}\left(N+1,\hbar +1\right)=\frac{y_{8}\left(N,\hbar +1\right)+u_{8}\left(N,\hbar +1\right)}{1+y_{8}\left(N,\hbar +1\right)}+8\cdot u_{8}\left(N,\hbar +1\right),$$

$$y_{9}\left(N+1,\hbar +1\right)=\frac{y_{9}\left(N,\hbar +1\right)+u_{9}\left(N,\hbar +1\right)}{1+y_{9}\left(N,\hbar +1\right)}+9\cdot u_{9}\left(N,\hbar +1\right).$$

**Figure 3:**
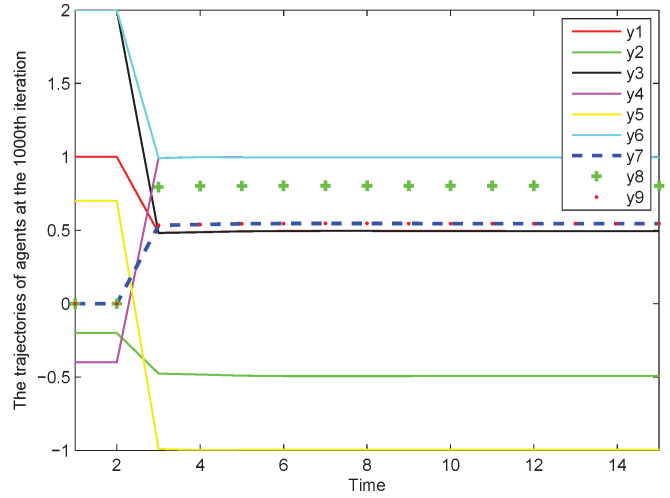
The output trajectories of MASs.

**Figure 4:**
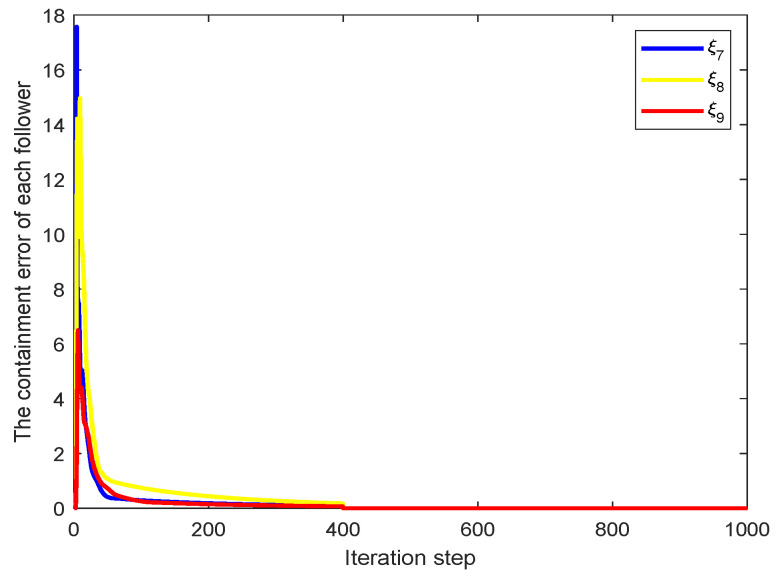
The containment error.

We choose ρ=0.1, λ=10, η=0.5, μ=1, $\sigma =10^{-5}$. It is worth noting that σ is the parameter used to set the reset condition of parameter ${\hat{\phi }}_{i}\left(N,\hbar \right)$. The initial state of each agent can be selected arbitrarily. Figure 3 shows that the bipartite containment task has been achieved. We can find that the output states of agents 1,3 converge to the same value $\frac{1}{2}$, and output state of agent 2 reaches a value with the opposite sign of agents 1,3. Similarly, the output states of agents 4,6 converge to the same value 1, and output state of agent 5 reaches a value with the opposite sign of agents 4,6. The output states of agents 7–9 asymptotically converge to the convex hull formed by agents 1–6. Figure 4 shows that the containment error of agents 7–9 asymptotically converges to zero as the number of iterations increases.

## 5. Discussion

The MFAILC is a control design method for nonlinear systems. Its basic idea is to establish an equivalent dynamic linear data model of each multi-agent system near each working point, and use the I/O data of the controlled system to estimate the partial derivatives of the system online. Then, the weighted one-step forward controller is designed by using the relationship between the MASs, and the MFAILC of nonlinear system is realized. Compared with the traditional adaptive ILC method, the model and algorithm proposed in this paper have some remarkable characteristics, as follows. First, the controller design only needs the I/O measurement data of the controlled system, without any model information. Therefore, traditional unmodeled dynamic problems do not exist under the MFAILC framework. Second, the MFAILC method has a simple structure and a small amount of computation. It does not require the construction of an accurate mathematical model of a multi-agent system, and any test signal and training process, thus it is a low-cost controller.

In the process of solving the bipartite containment problem of MASs, the existence of Assumption 1 has certain restrictions on the application of the MFAILC method. However, due to the limitation of communication bandwidth and storage space of MASs, the agent only transmits partial information. Therefore, Assumption 1 holds for some MASs. Moreover, the effect of unknown disturbances and time-varying network on the bipartite containment problem is not considered. We will solve this problem in the future. The simulations presented in this paper demonstrate the effectiveness of our proposed MFAILC method. In Figure 4, due to the selection of the initial value, the containment error of the followers is relatively large in the first few iterations. However, with the increase of the number of iterations, the containment error asymptotically converges to zero, that is, the bipartite containment control of the MASs is achieved by using the MFAILC method.

## 6. Conclusions

In this paper, the bipartite containment tracking problem for nonlinear MASs has been studied, where cooperative and antagonistic interactions between agents are considered. To solve this problem, we first show that, if the containment error converges to zero, then the bipartite containment can be achieved. Then, a MFAILC based on the dynamic linearization method is proposed. The designed controller only depends on the input and output data of MASs and does not need the model information of MASs. Furthermore, the condition that the containment error converges to zero is given, that is, all output states of followers asymptotically converge to the convex hull formed by the output states of leaders as well as leaders’ symmetric output states. The simulation verifies the effectiveness of the proposed method. Future studies will focus on the bipartite containment problem of MASs with unknown disturbances and time-varying network.

## Data Availability

Not applicable.
